# Buprenorphine for High-dose Tramadol Dependence: A Case Report of Successful Outpatient Treatment

**DOI:** 10.5811/cpcem.4914

**Published:** 2024-01-25

**Authors:** Leslie Mukau, Kadia Wormley, Christian Tomaszewski, Bushra Ahmad, Rais Vohra, Andrew A. Herring

**Affiliations:** * University of California San Diego, Department of Emergency Medicine, San Diego, California; † El Centro Regional Medical Center, Department of Emergency Medicine, El Centro, California; ‡ Highland Hospital-Alameda Health System, Department of Emergency Medicine, Oakland, California; § Imperial County Behavioral Health Services, Division of Substance Use Disorder Treatment Program, El Centro, California; ∥ University of California San Francisco-Fresno, Department of Emergency Medicine, Fresno, California; ¶ University of California San Francisco, Department of Emergency Medicine, San Francisco, California

We appreciate the interest expressed by the commentators with regard to our tramadol dependence case report.^1^ However, we respectfully disagree that not testing the tramadol product the patient obtained from Mexico nor testing the patient herself to confirm the presence of tramadol and exclude the presence of other opioids such as fentanyl is a major limitation of our case report.

We are well aware of the role increasingly being played by toxic adulterants and other pharmacologically active components, and specifically fentanyl, in illicitly manufactured pharmaceuticals in the United States and global street markets.^2^ ^,^ ^3^ These toxic adulterants either alone or in combination with other pharmacologically active components have been implicated as possible causes of adverse health outcomes, including death.^4^ Since 2021, illicit fentanyl has been involved in the vast majority of overdose deaths in the United States.^5^ Our patient was taking extremely high doses of tramadol and would most likely have overdosed and died had it been laced with fentanyl.

To expedite quick initiation of buprenorphine in the emergency department (ED) for patients with opioid use disorder (OUD), the California bridge model, increasingly adopted by many EDs in California and nationwide, does not encourage unnecessary treatment barriers such as diagnostic urine drug testing.^6^ At the Behavioral Health Outpatient Clinic, where the patient was originally seen, she had been referred for laboratory testing multiple times but did not go due to the coronavirus disease 2019 pandemic and behavioral changes due to her high-dose tramadol substance use disorder.

The majority of patients currently seen for OUD in most EDs are there due to fentanyl abuse, either deliberate or unintentional, and they do not usually have the symptoms that our patient was exhibiting. Seizures are usually a characteristic of high-dose tramadol use.^7^ Fentanyl, a synthetic μ-selective opioid agonist, which is typically 50–100 times more potent than morphine, does not cause seizures even at high doses. In fact, fentanyl in combination with certain neuroleptic medications as part of therapeutic neuroleptanalgesia can be used to treat seizures.^8^ ^,^ ^9^ As of this writing, our patient remains in treatment and not taking illicit tramadol and is currently free of seizures.

We, therefore, strongly believe our patient’s drug issues were most likely due to the use of high-dose tramadol.
